# Floral Morphogenesis: Stochastic Explorations of a Gene Network Epigenetic Landscape

**DOI:** 10.1371/journal.pone.0003626

**Published:** 2008-11-03

**Authors:** Elena R. Álvarez-Buylla, Álvaro Chaos, Maximino Aldana, Mariana Benítez, Yuriria Cortes-Poza, Carlos Espinosa-Soto, Diego A. Hartasánchez, R. Beau Lotto, David Malkin, Gerardo J. Escalera Santos, Pablo Padilla-Longoria

**Affiliations:** 1 Instituto de Ecología, Universidad Nacional Autónoma de México, Cd. Universitaria, México, D. F., México; 2 C3, Centro de Ciencias de la Complejidad, Cd. Universitaria, UNAM, México, D. F., México; 3 Instituto de Ciencias Físicas, Universidad Nacional Autónoma de México, Cuernavaca, Morelos, México; 4 Instituto de Investigación en Matemáticas Aplicadas y Sistemas, Universidad Nacional Autónoma de México, Cd. Universitaria, México, D. F., México; 5 lottolab, University College, London, United Kingdom; University of Calgary, Canada

## Abstract

In contrast to the classical view of development as a preprogrammed and deterministic process, recent studies have demonstrated that stochastic perturbations of highly non-linear systems may underlie the emergence and stability of biological patterns. Herein, we address the question of whether noise contributes to the generation of the stereotypical temporal pattern in gene expression during flower development. We modeled the regulatory network of organ identity genes in the *Arabidopsis thaliana* flower as a stochastic system. This network has previously been shown to converge to ten fixed-point attractors, each with gene expression arrays that characterize inflorescence cells and primordial cells of sepals, petals, stamens, and carpels. The network used is binary, and the logical rules that govern its dynamics are grounded in experimental evidence. We introduced different levels of uncertainty in the updating rules of the network. Interestingly, for a level of noise of around 0.5–10%, the system exhibited a sequence of transitions among attractors that mimics the sequence of gene activation configurations observed in real flowers. We also implemented the gene regulatory network as a continuous system using the Glass model of differential equations, that can be considered as a first approximation of kinetic-reaction equations, but which are not necessarily equivalent to the Boolean model. Interestingly, the Glass dynamics recover a temporal sequence of attractors, that is qualitatively similar, although not identical, to that obtained using the Boolean model. Thus, time ordering in the emergence of cell-fate patterns is not an artifact of synchronous updating in the Boolean model. Therefore, our model provides a novel explanation for the emergence and robustness of the ubiquitous temporal pattern of floral organ specification. It also constitutes a new approach to understanding morphogenesis, providing predictions on the population dynamics of cells with different genetic configurations during development.

## Introduction

“*All [the] epistemological value of the theory of probability is based on this: That large scale random phenomena in their collective action create strict*, *non random regularity*”. (From: B.V. Gnedenko and A.N. Kolmogorov, Limit Distributions for Sums of Independent Random Variables, Reading, Ma: Addison-Wesley, 1954).

The development of multicellular organisms consists of cell differentiation and spatiotemporal patterning. Since these processes arise from complex interactions among genetic and non-genetic elements, mathematical and computational models are useful to study the concerted action of these elements. Gene regulatory network (GRN) models, which are grounded in experimental data, have been able to recover fixed profiles of gene activation, that mimic those characterizing different cell types in both plants and animals (e.g., [1]–[3]). Such profiles correspond to the attractors of these networks, and have been interpreted as cell fates [4]–[7].

Some studies have explored cell-fate decisions by modeling transitions among attractors with stochastic gene regulatory networks (e.g. [8], [9]); however, models grounded in experimental data that are able to recover patterns of cell-fate attainment for a particular living system are only now starting to appear. Herein, we attempted to construct an integrative model driven by noise that explores the patterns of temporal cell-fate attainment in the experimental plant, *Arabidopsis thaliana* (L.) Heynh.

**Figure 1:**
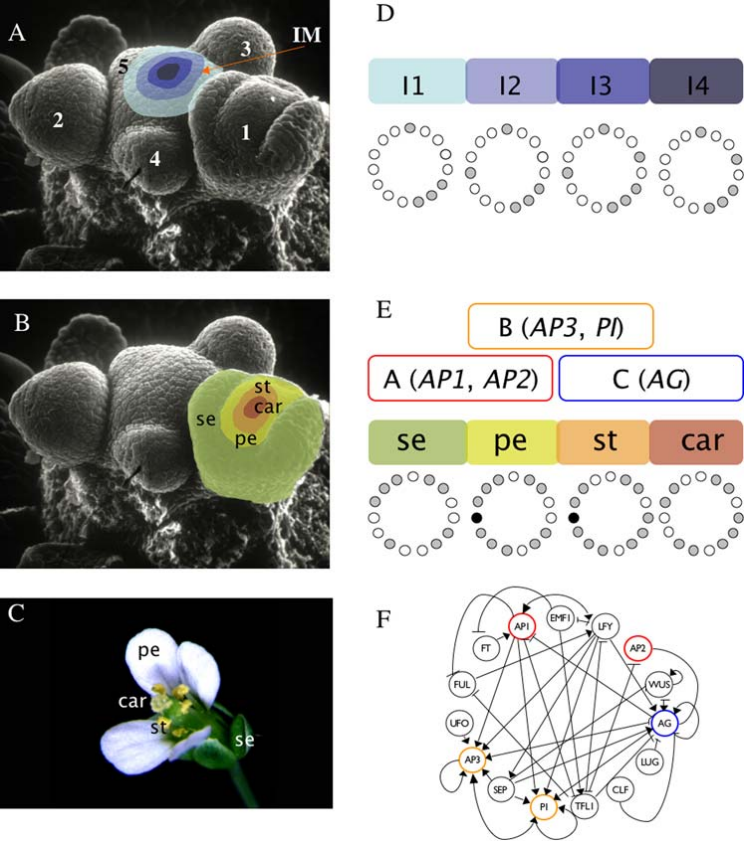
Flower development and gene network underlying primordial floral organ cell-fate determination in *Arabidopsis thaliana*. (A) The inflorescence meristem (IM in the Scanning Electron Micrography) is found at the apex of a reproductively mature plant. Within the IM, four regions can be distinguished. Interestingly, the experimentally observed gene activation configurations of each one of these regions are mimicked by the I1, I2, I3, and I4 attractors of the 15-gene GRN. Flower meristems arise in a helicoidal pattern from the flanks of the IM. The order in which floral meristems appear is indicated with numbers (1, oldest; 5, youngest). (B) Young flower meristems can be subdivided into four regions, each one containing the primordial cells that will eventually develop into the flower organs. In each floral meristem, the outermost region, which is first determined, will give rise to the sepal (se) primordium, the next to petals (pe) and finally, the primordial corresponding to stamens (st) and carpels (car) are determined in the center third and fourth whorls of the flower bud, respectively. (C) The mature flower of *Arabidopsis thaliana*. (D) I1, I2, I3, and I4 regions of the IM correspond to four of the attractors of the 15-gene GRN model. The expressed genes for each attractor are represented as gray circles, while the non-expressed genes correspond to white circles. (E) The other six attractors of the GRN model match gene expression profiles characteristic of sepal, petal (p1 and p2), stamen (st1 and st2), and carpel primordial cells. Black circles represent a gene (*UFO*) that can be either expressed or not expressed in the petal and stamen attractors, thus yielding two attractors for petal and stamen primordial cell-type. The gene activation profiles of the attractors recovered for the 15-gene GRN are congruent with the combinatorial activities of A, B, and C-type genes predicted by the ABC model of floral organ determination. See the Results section and [3], [12] for details. (F) Gene regulatory network model underlying cell fate determination in the IM and the flower meristem. A-genes (red), B-genes (yellow), and C-genes (blue) from the ABC model are indicated in the network.

In plants, morphogenesis takes place during the entire life cycle from groups of undifferentiated cells called meristems. Within meristems, cell fate is mostly determined by position rather than by cell lineage [10]. Flower meristems are formed from the flanks of the inflorescence meristem, which is found at the apex of an *Arabidopsis thaliana* plant once it has reached a reproductive stage (Figures 1A and B). Early in flower development, a floral meristem is sequentially partitioned into four regions, from which the floral organ primordia are formed and eventually give rise to sepals in the outermost whorl, then to petals in the second whorl, stamens in the third, and carpels in the fourth whorl in the central part of the flower (Figures 1B and C). This spatio-temporal sequence is widely conserved among the quarter of a million flowering plant species [11]; however, the dynamic mechanisms underlying this robust pattern are not yet understood.

In this study, we used a previously characterized Boolean GRN, which converges to ten attractors (Figure 1), to explore the dynamics of cell-fate decisions during the early stages of flower development. The ten attractors correspond to the main cell types observed during early flower development, namely, meristematic cells of the inflorescence, which is itself partitioned into four regions (I1, I2, I3, and I4; Figures 1A and D), and sepal, petal (P1 and P2), stamen (S1 and S2), and carpel primordial cells within flower meristems (Figures 1B and E) [3], [12]. This network was grounded in experimental data for 15 genes, wherein their interactions were formalized as logical functions. Among the 15 genes, five are grouped into three classes (A-type, B-type, and C-type), whose combinations are necessary for floral organ cell specification [13]. A-type genes (*AP1* and *AP2*) characterize sepal identity, A-type together with B-type (*AP3* and *PI*) petal identity, B-type and C-type (*AGAMOUS*) stamen identity, and the C-type gene (*AG*) alone for carpel primordia cell identity. The so-called ABC model describes such combinatorial activities during floral organ determination (Figures 1E and F) [13].

Different sets of initial conditions (basins of attraction) of the 15-gene regulatory network converge to the ABC-gene combinations necessary for floral organ determination [3], [12] (Figures 1E and F); however, this deterministic GRN does not enable studies of the transitions among the attractors. In this study, we investigated the temporal sequence with which attractors are visited in this GRN when noise or random perturbations to the output of the updating rules drive the system from one attractor to any other.

The obtained results demonstrate that noise alone is able to drive transitions among attractors with temporal patterns that mimic the sequence with which ABC-genes are activated (first A genes, then B genes, and finally the C gene) during early flower development [13]. These results are in line with the finding that the GRN in question is a robust developmental module that is widely conserved among flowering plant species [3]. Furthermore, the temporal cell-fate pattern during early stages of flower development seems to emerge from such a robust network in the presence of noisy perturbations. The results presented herein support the idea that random fluctuations in a system may be important for physiological adaptation, plasticity, and cell differentiation (examples in: [14]–[24]).

## Results

### A stochastic Boolean model of the GRN enables the study of transitions among network attractors

We first present the results obtained from the Boolean model of the GRN, and in the next section, we present the equivalent results obtained from a continuous model. The Boolean approach focuses on the state of genes' expression rather than on the concentration of their products. Thus, each gene in the network is represented by a Boolean variable *x* that takes the value *x* = 1, if the corresponding gene is expressed, and the value *x* = 0, if it is not expressed. The state of expression of the genes in the entire network (herein, configurations of the GRN, which correspond to “dynamic state of the network” used by some authors), is then represented by a vector with the set of Boolean variables {*x*_1_,*x*_2_,…,*x_N_*}, where *x_n_* is the state of expression of the *n^th^* gene and *N* is the total number of genes in the network. The state of expression of each gene changes in time according to the dynamic equation:
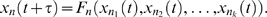
(1)


In the above equation, 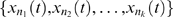 are the regulators of the gene *x_n_*, and *F_n_* is a Boolean function, also called a logical rule, which is constructed according to the combinatorial action of the regulators of *x_n_*. The additional parameter *τ* is a measure of the *relaxation time*, namely, of the time that it takes for a gene to change its state of expression under a change in the expression of its regulators. In the Boolean model, it is common to take *τ* = 1. Each gene in the network has its own associated Boolean function. This particular GRN includes 15 genes (Figure 1) whose logical functions are grounded in experimental biological data, as explained in [3]. The updated truth tables used here are available in [12].

Note that the dynamics given by Eq. (1) is deterministic: For a given set of Boolean functions, the configuration of the network at time *t* completely determines the configuration of the network at the next time step *t*+*τ*. Also note that since the number of dynamic states or configurations of the network is finite (Ω = 2*^N^*), under the dynamics given in Eq. (1), the network will eventually come back to a previously visited configuration, after which the network enters into a periodic pattern of expression. Such a periodic pattern is called an *attractor*, and all the initial configurations that eventually fall into that attractor constitute its *basin of attraction*. The deterministic version of the Boolean GRN modeled here recovered 10 fixed point attractors, each with a period equal to one, implying that the GRN remains in one of the 10 fixed 15-gene configurations after it reaches one of them.

Therefore, in the deterministic model defined in Eq. (1), once the system reaches an attractor, it remains there for all subsequent iterations; however, if noise is introduced into the logical rules, there is a finite probability for the system to “jump” from one basin of attraction to another. Our central aim herein was to address whether noisy perturbations of the logical rules in *A. thaliana* GRN are sufficient to recover the observed sequences of transitions among attractors (i.e., gene activity configurations characteristic of the primordial cell types within the floral meristem) during the development of this particular biological system.

The ten attractors of the 15-node GRN used here are as follows (Figure 1): Four corresponding to the four regions of the inflorescence meristem (I1, I2, I3, and I4), and six to the four floral organ primordial cells within the flower meristem (S, P1, P2, S1, S2, and C). The two attractors corresponding to petals (P1 and P2) are identical except for the state of activation of the *UFO* gene, and the same holds for the two stamen attractors (S1 and S2).

**Figure 2:**
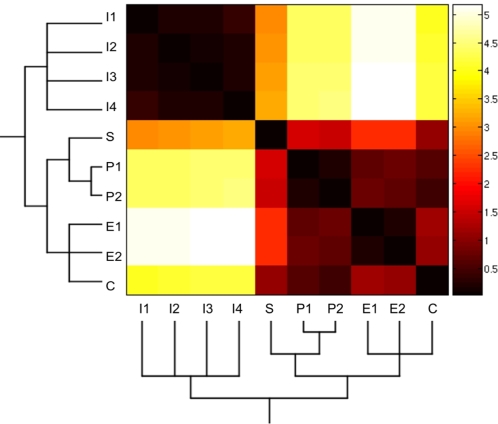
Heat map of the similarity matrix among the ten attractors of the GRN. A strict consensus phenogram was obtained for the GRN attractors (vectors of zeros and ones) by using the Manhattan distance similarity index (see Methods). This phenogram is shown below the attractors that are ordered along the X and Y axes of the heat map. Attractors that group together had the highest similarity indexes between them (i.e. the lowest Manhattan distance). Color scale: darker colors indicate more similar, while lighter ones indicate more different attractors in the pairs compared.

In the simulations of the stochastic versions of the GRN presented in this work, we did not consider the inflorescence attractors (I1–I4) because they are substantially separated from the floral primordia attractors. The distance between the two sets of attractors (inflorescence and floral) is clearly depicted by the way they are grouped in a phenogram (Figure 2). This is a branching diagram that groups entities according to their similarity (see Methods). The inflorescence meristem and floral organ primordia attractors cluster into two clearly distinct groups (Figure 2). Indeed, in simulations that considered all of the attractors, we found that, for a wide range of noise levels, the system never leaped out of the inflorescence attractors. On the other hand, when large noise magnitudes were considered, the system went from the inflorescence attractors to the carpel or stamen attractors, without visiting the sepal and petal attractors. Dismissing the I1–I4 attractors in the simulations allows for a better exploration of the temporal pattern in which the attractors corresponding to each of the four floral organ primordial cells are attained.

We used the GRN depicted in Figure 1 to examine which of the attractors (S, P1, P2, S1, S2, and C) the system is most likely to reach when it is initialized at a particular attractor and then is driven by noise to a different one. In order to obtain the transition probabilities among the different attractors (i.e., the entries of the so-called Markov matrix, see the detailed description below), the possible initial configurations of the system were exhaustively explored. Given any possible configuration (defined by an array of 15 entries with zeros and ones representing the activation states of the genes), the system was updated every iteration step according to the deterministic logical rules [12] with an error probability *η*. In other words, at each time step, each gene “disobeys” its Boolean function with a probability *η*, such that the dynamic rule in the presence of noise can be given by:
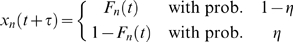
(2)


Note that the above equation reduces to Eq. (1) for *η* = 0. [In order to simplify the notation, we have written just *F_n_*(*t*) instead of 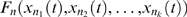.] These perturbations are applied independently and individually to each gene at each iteration.

If, after applying noise in one time step, the system remains in the same attractor or the same basin of attraction that it was before the noise was applied, one count is added to the main diagonal in the entry of the Markov matrix corresponding to that basin of attraction. If the configuration ended up in a different basin, a count is added to the row corresponding to the recipient basin in the Markov matrix (Table 1). This was repeated 10000 times for each of the Ω = 2*^N^* possible initial conditions. The number of realizations was fixed to a considerably larger number than that at which the matrix entries become stable (data not shown). The transition probabilities *P*(*n*|*m*) of the Markov matrix (Table 1) give the probability that a network in attractor *m* jumps to attractor *n* in the presence of noise, and are calculated by dividing the number of counts in each matrix entry by the total number of configurations that started in the corresponding matrix row.

**Table 1:** Markov matrix.

	sep	pe1	pe2	st1	st2	car
**sep**	0.939395	0.001943	0.009571	0.000083	0.00049	0.048517
**pe1**	0.036925	0.904162	0.00925	0.0339	0.000488	0.015275
**pe2**	0.009067	0.000464	0.941609	0.000024	0.048374	0.000461
**st1**	0.000084	0.001893	0.00002	0.936514	0.00996	0.05153
**st2**	0.00002	0.000001	0.002074	0.000356	0.987953	0.009597
**car**	0.002045	0.000034	0.00002	0.001951	0.01002	0.98593

Matrix of transition probabilities among all possible pairs of attractors. The entries of each column in this matrix correspond to the probabilities *P(n|m)* of reaching attractor n, given that the system is at attractor m at time *t = 0* (see Results and Methods, noise magnitude used for this case is 1%).

**Figure 3:**
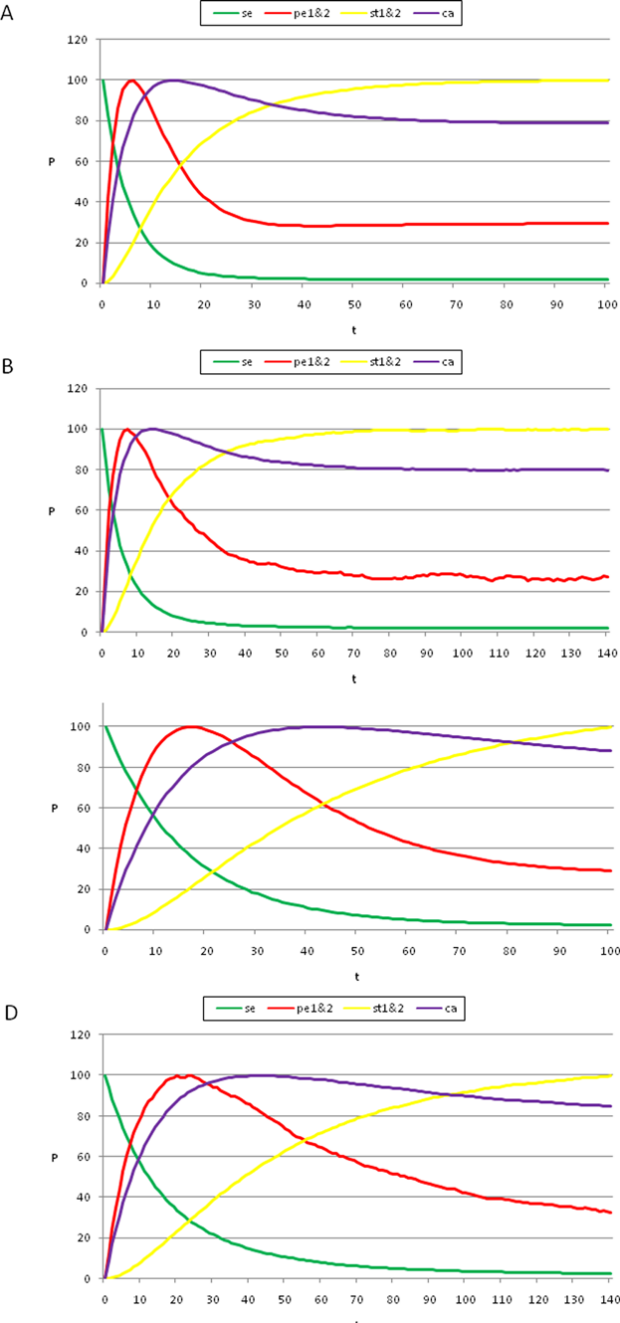
Temporal sequence of cell-fate attainment patterns under the Boolean dynamics with noise. Maximum relative probability (“Y” axis) of attaining each attractor, as a function of iteration number or time (“X” axis). (A) Probability of attaining each attractor (i.e., cell type) obtained by multiplying the Markov matrix M by a population vector 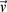 initialized at the sepal attractor. The error probability in computing this graph was η = 0.03. The most probable sequence of cell attainment is: Sepals, petals, carpels, and stamens. (B) Probability of attaining each attractor (i.e., cell type) at each iteration when 80000 randomly chosen “sepal” configurations were selected and followed for 140 steps. Noise was introduced in the updating of each gene independently, with a η = 0.03 probability at each iteration. The probabilities for the petal (p) and stamen (st) attractors correspond to the sum of p1+p2 and st1+st2, respectively. All maxima correspond to 100 because each absolute probability value was divided by the maximum of each attractor's curve (see Results and Methods). Equivalent graphs to those in (A) and (B) for *η* = 0.01 are shown in (C) and (D), respectively.

Since we wanted to find the most probable sequence of transitions among the attractors representing the various cell types, we followed the changes in the probability of reaching a certain attractor throughout time given that the system was initialized in a particular attractor at time *t* = 0 (see Figure 3). In order to achieve this, note that the Markov matrix (herein denoted as **M**) in Table 1 contains the conditional probabilities *P*(*n*|*m*) of reaching attractor *n* at time *t*+*τ*, given that the system is at attractor *m* at time *t*. In order to obtain the temporal sequence in which attractors are most likely reached, it is necessary to repeatedly multiply the Markov matrix **M** by the vector 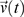, whose entries contain the fraction of cells at each attractor in a given population at time *t*. In other words, 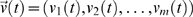, where *v*_1_(*t*) is the fraction of cells in the population whose configurations at time *t* are in the basin of attraction of the first attractor, *v*_2_(*t*) is the fraction of cells at time *t* in the basin of attraction of the second attractor, and so on. Starting out from a population with a given distribution 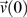 of cells among the attractors, the distribution of cells at time *t* is given by: 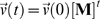.

Since we did not consider the four inflorescence attractors, only six attractors are involved in the dynamics. Therefore, **M** is a 6×6 matrix and 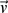 is a 6-dimensional vector. We also assumed that the total number of cells in the population always remains constant; hence, the sum of the six components of 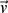 must sum to 100 (there are no “probability leaks” because transitions to the inflorescence attractors are extremely rare for the error levels used).

It is worth noting that the different attractors have basins of vastly different sizes. For instance, the basins of attraction of sepals and petals are very small in comparison to those of stamens and carpels. Therefore, the absolute probabilities for the attractors of sepals and petals are inevitably smaller than those of stamens and carpels; hence, in order to clearly observe the time at which each attractor attains its maximum probability, we divided each absolute probability value by the maximum of each attractor's curve, and plotted the relative probabilities for each attractor probability distribution. Note that since each curve was normalized in relation to its own maxima, the probabilities in these graphs no longer add up to 1 at every moment.

It is important to notice that although the Markov matrix **M** provides information about the probability of going from any attractor *m* at time *t* to any attractor *n* at time *t*+*τ*, this matrix alone is not sufficient to derive the most probable sequence of transitions among attractors. The latter is only evident when the matrix **M** is recursively multiplied by the vector 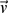 containing the fraction of cells per attractor, ideally until the system reaches a steady probability distribution.

Since sepal cells are the first to attain their fate in flower development, we used an initial vector 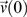 with *v*_1_(0) = 100 and *v_m_*(0) = 0 in all of the other entries (the first entry corresponds to the sepal configuration). Thus, initially, all of the population of cells within a floral primordia is in the sepal attractor. We then followed the changes in the probability of reaching each one of the other attractors over time, given that the entire system started in the sepal configuration (see Figure 3A). Every attractor has a maximum or peak in the probability of being reached at particular times. This maximum corresponds to the moment at which the corresponding primordial cell fate is most likely.

The use of the probability peaks to determine the time at which each cell multigenic configuration is most probable follows the standard reasoning in deriving maximum likelihood estimators in statistics [25]. The time at which the probability peak appears corresponds to the maximum of the associated transition probability for that particular attractor. The order of appearance of the peaks shown in Figure 3 matches the order of formation of the maxima of the transition probabilities. Recall that when using the maximum likelihood methodology [25], the main assumption is that the set of real data is precisely observed because they are more likely to happen than other possible data sets. In other words, they maximize the probability of being observed among all possible samples of the same size. Conversely, if we want to know when a specific event is more likely to happen, the most natural assumption is that it will be at a maximum of the corresponding probability distribution. This is precisely what we claim based on the graphs of the frequencies of visits to each attractor. Also notice that the locations of the maxima are not affected by normalization.

This interpretation hence implies that, given that a particular attractor will be reached (i.e. that a specific event will occur), it is natural to assume that the most likely time for it to occur is when the probability of reaching that particular attractor is maximal. Therefore, we propose that the temporal sequence in which attractors are attained will correspond to the sequence in which their maximum probabilities are reached.

A related important issue has to do with the interpretation of the transition probabilities. There are at least two possibilities that are consistent with the traditional approaches in statistical studies of collective behavior [26]. First, it is possible to consider that each agent (in this case, a single cell) will spend some time at each equilibrium configuration and then will jump to another with a certain probability. This would imply that each cell transits through different configurations. In our case, for example, a particular cell might attain a sepal primordia identity, then transit to a petal primordial cell, then to a stamen primordial cell, and finally to a carpel primordial cell. An alternative interpretation is that, from a given initial population of cells, the number of individual cells at a certain attractor at any given time, is proportional to the transition probability of reaching that particular attractor.

These two interpretations are equivalent or are assumed to be so (ergodic hypothesis) in many applications of statistical physics. This is often summarized by saying that averaging quantities in time is the same as averaging them in space [26]; however, in the case we have considered here, the second interpretation seems more appropriate. Future experimental studies that actually follow gene configurations over time at the individual cellular level will directly test these two alternative interpretations. For now, if we accept the overall population of undifferentiated cells in the floral meristem as our system, it is consistent to assume that the proportion of them reaching a particular configuration will be in accordance with the transition probabilities.

Therefore, we present a stochastic GRN that can be interpreted as a model of cell population dynamics. This model describes the dynamics of cells within the flower meristem, in which different fractions of cells sequentially attain distinct configurations. Therefore, it does not imply that individual cells transit through different identities or configurations, but rather that once in a floral meristem, one set of cells attains a certain identity first (sepal primordia) and then, from the remaining cells, another fraction attains a second cell fate (petal primordia), and so on, until all the cells in the floral primordium have reached an identity corresponding to each of the four floral organ primordia. Later in development, primordia will grow and differentiate to form the four floral mature organs: Sepals, petals, stamens, and carpels. The latter events are regulated by other GRNs. We explored whether the observed dynamics of cell-fate attainment can be recovered by the stochastic Boolean GRN model presented here.

### Simulated temporal transitions among attractors (cell types) mimicked the sequence in which A, B, and C genes are expressed in real flower meristems

By following the procedure presented above, we found that, by starting from the gene configuration associated with sepal primordial cells (t = 0 in Figures 3 A and C), the next maximum probability was observed in the petal curves, P1 plus P2 (t = 18 in Figure 3A). Afterwards, the peaks for the probability of attaining first the carpel and then the stamen (S1 plus S2) identity appeared (t≈45, t≈100 in Figures 3A and C). Interestingly, the same sequence was observed when applying a range of noise magnitudes from 0.5 to 10%; however, the peaks corresponding to the stamen and carpel cell fates became closer, almost simultaneous, as the noise magnitudes increased (compare Figures 3A–C). Nonetheless, it is noteworthy that the probability peak of the carpel configuration appeared before the peak of the stamen configuration.

The sequence resulting from the aforementioned model mimics the observed temporal pattern for A, B, and C gene expression: A-genes are expressed first, followed by B-genes, and finally by the C-gene [27], [28]. Furthermore, our model predicts that the gene configuration characteristic of carpels most probably appears before that corresponding to stamens during early flower development. This would, in fact, be the case if the C gene was first expressed in the flower center and then its expression expanded to the peripheral whorls. This should be tested experimentally by gathering data on the population dynamics of cells with different genetic configurations during early stages of flower development.

It is noteworthy that, among all of the tested noise levels, the only non-trivial temporal sequence of A, B, and C gene combinations recovered was: A, then AB, then C and finally BC. Although the latter two appeared almost simultaneously as error magnitudes used increased. This sequence is congruent with the ABC temporal pattern in *Arabidopsis thaliana* (Figures 3A and C) in which the A genes are turned on first, then the B and finally the C genes; hence BC and C combinations are defined at the same time. The trivial behaviors are: i) remaining in the initial configuration forever, and ii) transitions depending only on the size of the basins of attraction (i.e., the system behaves according to only noise). If the magnitude of the noise is increased, for example to 50%, the system goes from sepal to stamen1 or carpel configurations directly. This is because the basins of attraction corresponding to petals are very small in comparison to those of stamens and carpels.

In addition to the Markov matrix approach, we also performed simulations by directly following trajectories starting in randomly chosen configurations from the basin of attraction corresponding to the “sepal” configuration. We followed each of 80000 such configurations for 140 iterations in order to compute the probabilities of directly attaining each attractor at each iteration (see Methods). This latter simulation is directly comparable to that performed for the Glass system discussed in the following section. It is noteworthy that the sequence of probability peaks we found for each attractor over time is the same as the one that we had obtained using the Markov Matrix approach: Sepal, petal, carpel, and stamen (Figures 3B and D).

### Continuous GRN model with noise

In order to develop a continuous model based on the differential equations of the flower development GRN considered here, one would need to know all of the kinetic reaction constants, promoter affinities, degradation rates, and many other parameters involved in the dynamics. To the best of our knowledge, these have not yet been identified; however, a first step towards a continuous description of this GRN is to implement the Glass dynamics in the network [29]. This can be accomplished by considering the parameter *τ* in Eq. (1) as a small quantity, and expanding the left-hand side of that equation to the first order in powers of *τ*, which gives:
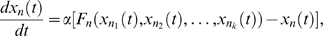
(3)where *α* = 1/*τ* is a measure of the “relaxation” time in the gene expression profile. Although the above equation is formally correct, it has the problem that the Boolean function *F_n_* on the right-hand side has to be evaluated using discrete variables, whereas the derivative on the left-hand side treats the *x_n_*'s as continuous variables. Therefore, each continuous variable *x_n_* has to be transformed into a discrete variable in order to evaluate the Boolean function. This is accomplished by introducing the discrete variables *xˆ* *_n_* defined as:
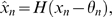
(4)where *θ_n_* is a threshold, and *H*(*x*) is the Heaviside function. (*H*(*x*) = 1 if *x*≥0 and *H*(*x*) = 0 if *x*<0). Thus, each continuous variable *x_n_*, representing the *level of expression* of a given gene, has an associated discrete variable *xˆ* *_n_* that represents the *state of expression* of that gene: “ON” if *x_n_* is above the threshold *θ_n_*, and “OFF” if *x_n_* is below *θ_n_*. In principle, each gene *x_n_* could have its own threshold *θ_n_*. Our simulations show that the results are qualitatively the same if we randomly assign the thresholds in the interval *θ_n_*∈[0.35,0.65]. Thus, in what follows, we fixed *θ_n_* = 0.5 for all of the genes.

The continuous piece-wise linear Glass dynamics of the network can thus be given by:
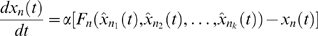
(5)


We will refer to the set of continuous values {*x*_1_(*t*),*x*_2_(*t*),…,*x_N_*(*t*)} as the *microscopic configuration* of the network, and to the set of corresponding discrete values {*xˆ*_1_(*t*),*xˆ*_2_(*t*),…,*xˆ* *_N_*(*t*)} as the *Boolean* configuration of the network. Note that there are infinitely many microscopic configurations compatible with the same Boolean configuration. Finally, we will refer to the dynamics generated by Eq. (5) as *Glass dynamics*.

It has been pointed out that the discrete model given in Eq. (1) and the corresponding continuous piece-wise linear model defined in Eq. (5) are not necessarily equivalent, since the attractors of the two models can be different, even when the Boolean functions *F_n_* are the same in both cases. Nonetheless, our numerical simulations show that for the *A. thaliana* network, the Glass dynamics generate exactly the same ten point attractors obtained in the Boolean model, and only those ten attractors. Therefore, from now on, we will make no distinction between the attractors of the Boolean model and the attractors of the continuous model, referring to them simply as the attractors of the floral GRN.

**Figure 4:**
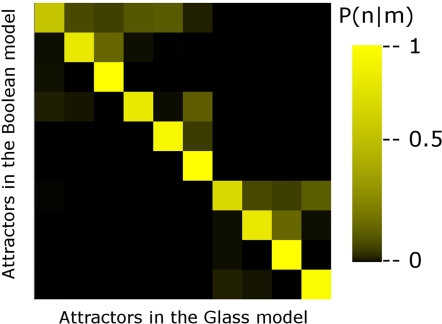
Changes in the basins of attraction of the continuous model with respect to the Boolean model. Color map of the probability *P*(*n*|*m*) that a microscopic configuration whose associated Boolean configuration belongs to the basin of attraction of attractor *m*, ends up in attractor *n* using Glass dynamics. Note that the main transitions occur along the diagonal where attractors are reached by both dynamics (Boolean and Glass); however, the non-diagonal elements indicate that two microscopic configurations that correspond to the same Boolean configuration may end up in different attractors.

Even when the Boolean dynamics and the Glass dynamics produce the same ten attractors, their basins of attraction do change from one model to the other. This is so because two different initial microscopic configurations that correspond to the same Boolean configuration may end up in two different attractors under the Glass dynamics. In order to show that this is indeed the case, for each of the Ω = 2*^N^* Boolean configurations of the network, we probed 10,000 compatible microscopic configurations. We evolved these 10,000 microscopic configurations in time until an attractor was reached, and determined the configuration in which the network fell. Figure 4 depicts in a color map the probability *P_G_*(*n*|*m*) that the network ends up in attractor *n* under the Glass dynamics, given that it started in a microscopic configuration whose corresponding Boolean configuration was in the basin of attraction of attractor *m*. As can be seen, the highest probabilities lie along the diagonal; however, the non-vanishing off-diagonal elements indicate that two different microscopic configurationss corresponding to the same Boolean configuration may end up in two different attractors.

On the other hand, Table 2 shows the fractional sizes of the basins of attraction in both the Boolean and the continuous models. It is apparent from this table that, when passing from the Boolean to the continuous description, the largest basins of attraction (carpel and stamen1) lose about 30 to 40 percent of their configurations, which are redistributed among the smaller basins of attraction. Thus, even when the predicted cell types (attractors) are the same in the two models, the basins of attraction are not.

**Table 2:** Basins of attraction.

Attractor	Boolean Dynamics	Glass Dynamics
Inflorescence 1	0.0156	0.0500
Inflorescence 2	0.0156	0.0500
Inflorescence 3	0.0078	0.0380
Inflorescence 4	0.0078	0.0381
Carpel	0.4404	0.2622
Sepal	0.0185	0.0670
Stamen 1	0.4570	0.3331
Stamen 2	0.0166	0.0710
Petal 1	0.0195	0.0786
Petal 2	0.000976	0.0116

This table shows the fractional sizes of the basins of attraction in the Boolean and Glass models. The data for the Glass dynamics were obtained by sampling 10,000 microscopic configurations for each of the Ω = 2*^N^* Boolean configurations, and by counting the frequency with which these microscopic configurations end up in each of the ten attractors.

### The stochastic continuous model of the GRN yields a cell-fate attainment sequence similar to the Boolean stochastic model

In order to implement noise in the continuous model, we followed a procedure similar to the one indicated in Eq. (2); namely, with a probability *η*, each gene will disobey its Boolean function *F_n_*, replacing it by 1−*F_n_*; however, since the system in this case is governed by differential equations, this “perturbation” will occur during a *finite time interval* Δ*t_p_*, rather than being instantaneous. In other words, if at time *t* one particular gene *x_n_* is perturbed and chosen to disobey its Boolean function, then from time *t* to time *t*+Δ*t_p_* its state will not be determined by Eq. (5), but rather by the equation:
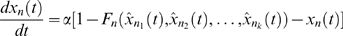
(6)


After the time interval Δ*t_p_*, the state of *x_n_* will be determined again by Eq. (5), and a new set of “disobeying genes” will be chosen. We will call these disobeying genes the *perturbed genes*.

**Figure 5:**
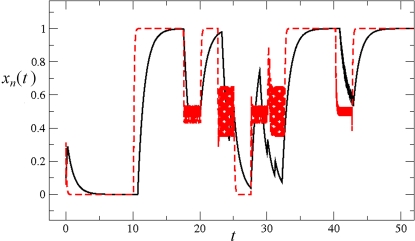
Effects of the choice of the relaxation time on Glass dynamics with noise. Two typical realizations of Glass dynamics for a given gene *x_n_* showing that the choices of the relaxation time *τ* and the perturbation time Δ*t_p_* do not affect the qualitative dynamics, so long as Δ*t_p_*>*τ*. Both trajectories started from the same initial conditions, and were followed through the same set of perturbations. The black trajectory corresponds to Δ*t_p_* = 2.5 and *τ* = 1, whereas the red trajectory corresponds to Δ*t_p_* = 2.5 and *τ* = 1/20.

**Figure 6:**
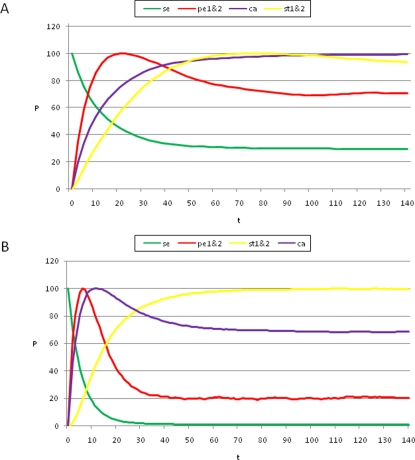
Temporal sequence of cell-fate attainment patterns under the Glass dynamics with noise. Maximum relative probability (“Y” axis) of attaining each attractor as a function of iteration number or time (“X” axis). (A) The maxima of the cell-fate curves are attained in a particular sequence in time, which in this case is sepal, petal, stamen, and carpel. Parameters used: dt = 0.01, τ = 1, and Δ*t_p_* = 2.5. (B) When the simulations mimic the Boolean case (dt = 1, τ = 1 and Δ*t_p_* = 1; see Results and Methods), a temporal pattern identical to that of the Boolean dynamics was obtained, with a sequence of sepal, petal, carpel and stamen. The noise used in both cases was η = 0.03. Although the Boolean and Glass dynamics need not coincide in general, for the case of the *A. thaliana* GRN, both models provide similar predictions. Simulations show that the order of emergence of the stamen and carpel maxima, as compared to the Boolean model, may depend on the precise values of the kinetic constants.

We have to choose the value of Δ*t_p_* in such a way that the gene has enough time to relax to its new state after the perturbation has been produced. In other words, Δ*t_p_* has to be larger (or at least of the same order of magnitude) than the relaxation time *τ* = *α* ^−1^ appearing in Eq. (5). Figure 5 shows two typical noisy realizations of the temporal evolution of a particular *x_n_*(*t*) as a function of time, for two different choices of *τ* and Δ*t_p_*: One for Δ*t_p_* = 2.5 and *τ* = 1 (black curve), and the other for Δ*t_p_* = 2.5 and *τ* = 1/20 (red curve). The two realizations started out from the same initial conditions, and underwent the same set of perturbations. The only difference was the value of *τ*. As can be seen from this figure, the trajectories are qualitatively the same as long as Δ*t_p_*>*τ*. In what follows, we selected Δ*t_p_* = 2.5 or 1 (Figures 6A and B, respectively), and *τ* = 1 to simulate Glass dynamics with noise (see methods for further details).

In order to determine the cell-fate attainment patterns in the *A. thaliana* network under Glass dynamics with noise, we analyzed the transitions between attractors over time in a population of 80 000 cells subject to the perturbations described above. At time *t* = 0, all of the cells were initialized in different random microscopic configurations corresponding to the sepal basin of attraction. In every cell, each gene was independently chosen to be perturbed with a probability *η* = 0.03. The non-perturbed genes then evolved in time according to Eq. (5), whereas the perturbed genes evolved following Eq. (6). After a time interval Δ*t_p_* = 2.5 or 1 (for Figures 6A and B, respectively), a new set of perturbed genes in the entire population was chosen again, and so on.

At each unit of time, we looked at the microscopic configuration of each cell and determined to which attractor this microscopic configuration would have evolved in the absence of perturbations. This allowed us to associate a given attractor at each unit of time to each cell configuration. The results of this simulation are reported in Figure 6, which shows the evolution in time of the population of cells, and shows how the cells redistribute among the sepal, petal, stamen, and carpel attractors. Similar patterns were recovered for other values of noise (data not shown), ranging between *η* = 0.005 and *η* = 0.1, as well as for other values of Δ*t_p_*.

As can be observed in Figure 6, the results obtained using Glass dynamics are analogous to those obtained for the Boolean model, in that the addition of noise to the dynamics produces the emergence of cell-fate attainment patterns in a population of cells in a specific temporal order. Thus, the use of the Glass model, based on piece-wise linear differential equations, reveals that the time ordering in the emergence of the cell-fate patterns is not an artifact of the synchronous updating in the Boolean model; however, the stamen and carpel peaks are reversed in time between the Boolean and Glass models (Figure 3 vs. Figure 6A).

In real flowers, A genes are first “ON”, followed by the B genes that turn “ON,” thus defining the A (sepal) to AB (petal) transition. This is recovered by both models (Figures 3 and 6), and is observed in real flowers. The C genes then turn “ON,” and hence, the BC (stamens) and C (carpels) configurations are defined at the same time. While the Boolean dynamics predict that the carpel primordia cell fate (C alone) will be attained before that of the stamen (BC), in the Glass model, these two are reversed (Figure 3 vs. Figure 6A). Interestingly, when this model is simulated to mimic the Boolean model (Figure 6B), both systems recover the same sequence: “Sepal-petal-carpel-stamen” (Figure 3 vs. Figure 6B) and in both cases the time at which stamen and carpel configurations are determined converge as noise levels are increased. Detailed experimental data on the precise spatio-temporal dynamics of the gene activation profiles of cells in the developing flower meristem are needed to test which of the two peaks is observed first in real floral buds. Such data will also be useful to determine which of the two models predicts the most realistic frequency distributions of cell types over time. The latter will be related to the relative sizes of the basins of attraction.

Glass system simulations indicate that the order of appearance of the two peaks (stamen or carpel) may depend on the precise values of the reaction-kinetic constants and degradation times, as well as some other epigenetic processes not taken into consideration in the simple analysis presented here. The important conclusion of both models is that noise in the gene-expression dynamics is necessary and sufficient to qualitatively recover the temporal transitions among the ABC-gene configurations observed during early flower development.

## Discussion

Robust morphogenetic patterns that are recreated over the life cycles of individuals from the same species, or even from distantly related species, have led to the prevailing view of development as a deterministic process; however, we have shown here that the stereotypical temporal pattern with which floral organs are determined may result from a stochastic dynamic system associated with a highly non-linear GRN.

This study supports recent work that has concluded that random fluctuations in a system may be important for cell behavior and pattern formation ([14]–[21]), and contrasts with deterministic and preprogrammed views of development. Intrinsic noise (noise arising from the system itself) has its origin in molecular fluctuations due, for example, to slight modifications in temperature, and in random events due to sampling, given that the number of molecules is not infinite during transcription and translation [16], [19], [14].

**Figure 7:**
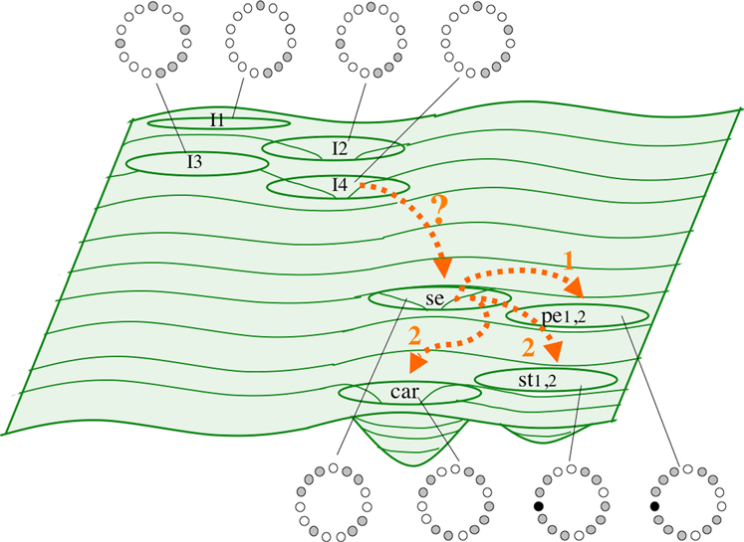
Schematic representation of the epigenetic landscape generated by a stochastic exploration of the GRN for flower development. This schematic landscape is equivalent to the Epigenetic Landscape proposed by C.H. Waddington (1957). Basins comprise the cell genetic configurations that lead to attractors (in this case, gene arrays characteristic of floral organ primordial cell-types: Sepals, petals, stamens, and carpels. See Figure 1 and Discussion). Each cell fate is associated to the GRN configuration corresponding to each of the attractors. The arrows represent transitions among attractors. The transition from inflorescence to sepal attractor might be biased or determined by an inducer. The numbers associated to the arrows represent the sequence of transitions among attractors: From sepals to petals, and then to carpels and stamens.

Stochastic implementations of a GRN model as pursued in this study were proposed by C. H. Waddington many years ago ([22]; see review in [23]). He understood development as a complex dynamic system, with genes, proteins, metabolites, and environmental factors constituting complex dynamic networks. The attractors of such networks represent a specific configuration of the system (e.g. cell types). The number, depth, width, and relative position of these attractors are represented by the hills and valleys of his “Epigenetic Landscape” metaphor [22], [7]. The study presented here actually explored such an Epigenetic Landscape for the flower organ determination GRN (Figures 1 and 7). Other recent studies have also explored this idea for GRNs [30].

In the case presented here, a GRN generates the overall temporal morphogenetic pattern (Figures 3 and 6) observed during flower development of *Arabidopsis thaliana* [31], [32]: A genes are expressed first, followed by B genes, and finally C genes, in a rather broad range of noise magnitudes, and in two different modeling approaches. Therefore, our results provide a possible explanation for the conservation, among many flowering plant species [27], [28], [31]–[34], of the temporal transitions of A, B, and C-gene expression, and to some extent, of the observed cell fate attainment patterns.

Our results support the hypothesis that biological systems may not only cope with random perturbations, but that the noise may have been incorporated during evolution in the generation of biological patterns (*e.g.* [30], [35]–[37]). Central to the constructive role of noise is the existence of non-linear dynamic systems [38] that converge to robust attractors for a range of noise magnitudes. Stochastic implementations of GRNs, such as the one presented here, may guide predictions of actual noise magnitudes experienced in biological systems.

Nevertheless, deterministic signals or inducers of flower development cannot be dismissed. Indeed, our results hold when focusing on the attractors corresponding to the four types of floral organ primordia. However, if all of the attractors (including I1–I4) are considered, and the system is initialized in one of the inflorescence basins, the system hardly ever transits into the floral basins when small noise levels are used, or else it directly jumps to one of the largest basins (stamens1 or carpels) when larger magnitudes of noise are simulated. These results enable us to speculate on the role of reported non-random inducing signals in the transition from cell fates in the inflorescence meristem to those in the flower meristem. Genes such as *FLOWERING LOCUS T*, *SUPPRESSOR OF OVEREXPRESSION OF CO 1*, or *CONSTANS* (see [39] for a review) could constitute or mediate such signals.

The type of model put forward here will enable the predictions of the real magnitudes of stochastic fluctuations once such deterministic biasing signals are considered. They will also be useful to test what mutations may cause alterations in the epigenetic landscape and alter the temporal order with which attractors are visited. Such models will guide the search of genetic alterations underlying atypical morphogenetic patterns during the evolution of flowering plant species [40].

One possible interpretation of our model is to assume that, once most cells have attained a certain attractor within a primordium, these are canalized to develop into a particular organ type. One possible explanation for this is that noise does not drive the cells out of each configuration once a certain proportion of them attain an attractor, or that the noise is “frozen” at some point, maybe because irreversible differentiation or synchronization events take place. We may speculate that, in the developmental system we have studied, non-autonomous cell function of key transcription factors [41]–[44] could play a relevant role in this process, as it could effectively freeze the stochastic fluctuations or synchronize the configuration of the cells within a primordium, and thus, contribute to the formation of the observed spatio-temporal patterns. We could further speculate that the activity of pre-patterning genes (e.g., *WUSCHEL* or *UNUSUAL FLORAL ORGANS*; [43], [45], [46]) may play important roles during spatio-temporal pattern formation.

Models such as those presented here enable novel predictions about the genetic regulation of cell differentiation and morphogenetic patterns. For example, the stochastic GRN dynamic system eventually attains a stationary distribution of attractor probabilities. The distribution reflects the probability of the cells being in each attractor, and may be interpreted as the proportion of primordial cells fixed to each GRN configuration. In the floral organ specification network, such proportions would correspond to the regions within the floral meristem with A, A+B, B+C, and C function configurations; however, this distribution may only be observed at the very early stages of the partitioning of the floral bud into four concentric rings. This event occurs before cells committed to a certain cell-type start further differentiation and acquire distinct division and elongation rates; hence, the final amount of cells in a certain organ or organ primordium would not necessarily coincide with that predicted by the models presented herein.

Another prediction derived from this model states that the carpel attractor appears either before (Figure 3) or after (Figure 6A) that of stamens. This prediction does not contradict the fact that, in most plants, carpels are the last organs to be fully formed because, again, cells have different division and elongation rates after cell-type differentiation, and therefore, the order in which organogenesis takes place may not match the sequence in which organ primordia cells are determined during early flower development, before the primordia actually emerge.

The discussion above suggests that models that incorporate GRN associated to cellular growth and proliferation, as well as spatial aspects of the system presented here, will eventually be needed to understand the dynamics by which cells attain their fate and proliferate in the floral spatio-temporal domain. In this paper, we have restricted ourselves to exploring the temporal patterns of cell-fate establishment early in flower development, assuming that cells differentiate independently of one another; however, in real organisms, cell-cell communication, cellular dynamics, domain geometry, and growth or mechanical interactions, are all likely to alter the proportion of cells across space and time that are set aside for each type in early flower development [10].

Kauffman's Boolean model for cell differentiation has been criticized because it is said to oversimplify the gene regulatory interactions and the way activation states of all genes are updated (synchronically in Kauffman's proposal); however, Boolean GRN models grounded in experimental data have been able to recover observed multi-gene expression arrays characteristic of certain cell types in several biological systems [2], [3], [7], [36]. These results suggest that the logic of regulation considered in Boolean networks suffices to qualitatively reproduce the dynamics of biological GRNs. Furthermore, theoretical studies have suggested that the details of the kinetic functions are not relevant in determining the system's attractors. In particular, updating schemes do not seem to affect the number and identity of fixed-point attractors [47], as is the case of the attractors recovered in the network used here.

Given that the identity of the attractors and the temporal sequence in which these were attained are the same (Figures 3A–C vs Figure 6B) or very similar (Figures 3A–C vs Figure 6A) using Boolean and Glass dynamics, this study reveals that the time ordering in the emergence of cell-fate patterns is not an artifact of synchronous updating in the Boolean model; however, the sizes of the basins of attraction differ between the two models. In Glass dynamics, the basins corresponding to stamen and carpel primordia cells are smaller, and those of sepals and petals are larger (Table 2); hence, the proportion of cells at each fate along time predicted by the Glass and Boolean dynamics differ, which suggests that the updating schemes might be relevant to recovering the actual temporal cell population dynamics in biological systems. Experimental data on the temporal fluctuations of primordial cells with different multi-gene expression arrays will test which of the two systems and updating hypotheses better reproduces the real system.

Eventual formalizations of stochastic multicellular GRN dynamics in explicit spatial domains may require “hybrid” approximations that enable large computational explorations, and allow, for instance, the explicit incorporation of developmental processes into models of network or phenotypic evolution [48], or the study of the epigenetic landscapes that emerge from GRN related to complex diseases, such as cancer [9].

In conclusion, we put forward a stochastic approach to model the Boolean and continuous dynamics of an experimentally-based GRN, and thus, take Waddington's Epigenetic Landscapes into a specific biological framework: Flower organ specification in *Arabidopsis thaliana*. The theoretical framework of this proposal could also be useful for studying the behavior of other networks, including, for instance, ecological, epidemiological, immunological, engineering, or social networks. Finally, our results emphasize that complex networks and stochastic processes are central to understanding the biological development and emergence, as well as the stability, of morphogenetic patterns.

## Methods

### Construction of phenogram of attractors

We obtained six phenograms by estimating the Manhattan distance index to infer the relationships among the 10 attractors for the 15-gene system. This index was obtained by comparing the vectors of zeros and ones of each attractor. We then used the clustering method by the unweighted pair-group method with arithmetic average (UPGMA) to group the attractors. We obtained six different phenograms, with which we constructed a strict consensus that kept the branches that were recovered in all of the six phenograms. In Figure 2, the consensus phenogram is shown below the attractors ordered along the X and Y-axes of the heat map, corresponding to the Similarity Matrix.

### Implementation of noise in the GRN model

#### Boolean case

The GRN has 15 elements; two of them (LUG and CLF) are constitutively expressed in the flower meristem, and thus, their activation states were fixed to 1. The transition probabilities among attractors in the Boolean GRN implementation were obtained by introducing noise to the updating logical rules in 10, 000 realizations for each possible configuration of the system. The analyses of the Boolean model were performed with the “Atalia” software, which is publically available (http://www.ecologia.unam.mx/~achaos/Atalia/atalia.htm).

Another equivalent method to obtain the Markov matrix entries would be to follow the system's trajectory for every possible initial configuration. For certain levels of noise, the system never remains at a particular basin, and it is hard to determine when to stop the computation for the corresponding initial condition. Nonetheless, we performed a similar type of simulation in order to mimic that of the Glass system. We selected a random configuration from those in the “sepal” basin. Each gene was updated according to its true table, except that with a certain probability (0.01 and 0.03), the rule was violated, and if the true table predicted that a state should be “1,” it was set to “0,” and vice versa. The new basin was registered, and this procedure was continued for 140 iterations. 80,000 such realizations were obtained (i.e., 80,000 randomly chosen configurations from the “sepal” basin were chosen).

#### Glass system

The model is explained in the Results section. We numerically integrated the set of differential equations (5) and (6) using the Euler method with an integration step *dt* = 0.01. The results do not change by choosing smaller values of *dt*; however, if we take *dt* = τ = Δ*t*_p_ = 1, then the continuous model given in equations (5) and (6) becomes completely equivalent to the Boolean model given in Eq. (2). The results for this latter case are shown in Figure 6B. In order to recover the temporal sequence, in which attractors (cell-fate) were attained in the *A. thaliana* network using Glass dynamics with noise, we followed transitions for 140 time-steps, starting with a population of 80 000 cells (configurations from the “sepal” basin of attraction), in which each gene was independently chosen not to be updated according to its logical functions (set to “1” if the predicted value was “0,” and vice versa), with a probability *η* = 0.03; hence, the non-perturbed genes evolved in time according to Eq. (5), while the perturbed genes evolved following Eq. (6). After a time interval Δ*t_p_* = 2.5 for Figures 6A, and 1 for Figure 6B, a new set of perturbed genes in the entire population was chosen again, and so on until 140 iterations were completed. Qualitatively similar results were obtained for a noise of 0.01. The code for the Glass system simulations was developed in JAVA, and is available upon request.
